# Genome-wide mapping of signatures of selection using a high-density array identified candidate genes for growth traits and local adaptation in chickens

**DOI:** 10.1186/s12711-023-00790-6

**Published:** 2023-03-23

**Authors:** Salvatore Mastrangelo, Slim Ben-Jemaa, Francesco Perini, Filippo Cendron, Filippo Biscarini, Emiliano Lasagna, Mauro Penasa, Martino Cassandro

**Affiliations:** 1 Department of Agricultural, Food and Forest Sciences, University of Palermo, 90128 Palermo, Italy; 2 Laboratoire des Productions Animales et Fourragères, Institut National de la Recherche Agronomique de Tunisie, Université de Carthage, 2049 Ariana, Tunisia; 3 Department of Agricultural, Food and Environmental Sciences, University of Perugia, 06121 Perugia, Italy; 4 Department of Agronomy, Food, Natural Resources, Animals and Environment, University of Padova, 35020 Legnaro, Italy; 5 Institute of Agricultural Biology and Biotechnology (IBBA), National Research Council (CNR), 20133 Milan, Italy; 6 Federazione delle Associazioni Nazionali di Razza e Specie, 00187 Rome, Italy

## Abstract

**Background:**

Availability of single nucleotide polymorphism (SNP) genotyping arrays and progress in statistical analyses have allowed the identification of genomic regions and genes under selection in chicken. In this study, SNP data from the 600 K Affymetrix chicken array were used to detect signatures of selection in 23 local Italian chicken populations. The populations were categorized into four groups for comparative analysis based on live weight (heavy vs light) and geographical area (Northern vs Southern Italy). Putative signatures of selection were investigated by combining three extended haplotype homozygosity (EHH) statistical approaches to quantify excess of haplotype homozygosity within (*iHS*) and between (*Rsb* and *XP-EHH*) groups. Presence of runs of homozygosity (ROH) islands was also analysed for each group.

**Results:**

After editing, 541 animals and 313,508 SNPs were available for statistical analyses. In total, 15 candidate genomic regions that are potentially under selection were detected among the four groups: eight within a group by *iHS* and seven by combining the results of *Rsb* and *XP-EHH*, which revealed divergent selection between the groups. The largest overlap between genomic regions identified to be under selection by the three approaches was on chicken chromosome 8. Twenty-one genomic regions were identified with the ROH approach but none of these overlapped with regions identified with the three EHH-derived statistics. Some of the identified regions under selection contained candidate genes with biological functions related to environmental stress, immune responses, and disease resistance, which indicate local adaptation of these chicken populations.

**Conclusions:**

Compared to commercial lines, local populations are predominantly reared as backyard chickens, and thus, may have developed stronger resistance to environmental challenges. Our results indicate that selection can play an important role in shaping signatures of selection in local chicken populations and can be a starting point to identify gene mutations that could have a useful role with respect to climate change.

**Supplementary Information:**

The online version contains supplementary material available at 10.1186/s12711-023-00790-6.

## Background

When chicken were first domesticated and spread is still a matter of debate. The jungle fowl has been suggested as the first domesticated chicken, through multiple independent events [1] that took place in a relatively short evolutionary time [2], although a recent study has raised questions about this assumption [3]. After domestication, natural and artificial selection led to different strains of chickens, which are clustered into breeds or populations based on their phenotypic characteristics and the environmental conditions in which they are reared. Several chicken breeds have been strongly selected for meat or egg production, and since the twentieth century, this selection has led to commercial breeds or lines with high performances. However, other chicken breeds (e.g., local populations) have not been selected for production and for which natural selection and genetic drift are the major driving forces for shaping their pattern of genetic variation.

Selection is responsible for changes in specific genomic regions called “signatures of selection”, which have a role in traits related to e.g. morphology, production, immune-response, and adaptation to different environments [4]. Investigating the presence of signatures of selection is important to better understand the evolutionary history of livestock populations and the genetic mechanisms that underlie phenotypic differentiation [5]. Moreover, signatures of selection can be used to identify genes that exert an advantage for a particular population [6].

A previous study that used a massively parallel sequencing approach discovered genomic regions and genes that may have been selected during chicken domestication and selective breeding [7]. However, this study included only a few breeds and the overall selection history for domesticated and wild chickens remains unclear.

Availability of single nucleotide polymorphism (SNP) genotyping arrays and progress in statistical analysis have allowed the identification of genomic regions and genes that have undergone positive selection in chicken [8–11]. Different approaches have been proposed for the identification of signatures of selection, which include statistical methods that are based on linkage disequilibrium (LD), differences in allele frequency, homozygosity regions, and haplotype structure. As recombination does not (or seldom) occur during the rapid increase in the frequency of a haplotype that bears a beneficial mutation, an ongoing or incomplete signature of selection contains a high-frequency haplotype with broad LD. Relative extended haplotype homozygosity (EHH)-derived statistics [12] are the most efficient to identify higher-homozygosity regions with greater accuracy than single allele frequency approaches. Among these EHH-derived statistics, the most common are (i) the integrated haplotype score (*iHS*), which is a within-population test [13], (ii) the standardized log-ratio of the integrated site-specific EHH between pairs of populations test (*Rsb)* [14], and (iii) the cross-population EHH test (*XP-EHH*) [15]. Selection also leads to reduced genetic diversity in some regions of the genome, which results in stretches of consecutive homozygous genotypes, known as runs of homozygosity (ROH) islands. Previous studies have shown that ROH islands can be used to identify genomic regions that affect production or adaptation in livestock [16–18].

According to the FAO (DAD-IS), the conservation status of several local Italian chicken breeds can be regarded as critical. Preliminary analyses on the genetic diversity and population structure of local Italian chicken populations have already been reported using genome-wide SNP data. The patterns of genetic differentiation showed that most of these populations formed non-overlapping clusters and were separated. In addition, some populations showed low effective population sizes and high levels of inbreeding, resulting in risk of extinction [19]. However, to date, no comparative genome-wide search for signatures of selection has been conducted in these chicken populations.

In the present study, genome-wide information from the 600 K Affymetrix chicken SNP array and *iHS*, *Rsb, XP-EHH,* and ROH approaches were used in comparative analyses of local Italian chicken populations to detect signatures of selection and unravel the effect of selection and environmental pressure on these important local genetic resources.

## Methods

### Animal samples and quality control

All animals were genotyped using the Affymetrix Axiom 600 K Chicken Genotyping Array (for full details see Cendron et al. [19]). The data were edited using the PLINK 1.9 software [20] to remove SNPs with a call rate lower than 95%, SNPs with a minor allele frequency lower than 5%, and animals with more than 10% missing genotypes. To avoid multicollinearity effects, the genotype data were subjected to LD pruning using the PLINK 1.9 software [20], with a SNP window size of 50, step of 5 SNPs, and *R*^2^ of 0.60. After editing, genotypes on 541 animals from 23 local Italian chicken populations (Table 1) and for 313,508 SNPs remained.

**Table 1:** List of chicken breeds and number of animals (N) that composed the heavy/light and Northern/Southern groups

Breed	Code	N	Population group			
			Heavy	Light	Northern	Southern
Ancona	ANC	24				
Bianca di Saluzzo	BSA	24			X	
Bionda Piemontese	BPT	22			X	
Cornuta Caltanissetta	COR	22				X
Ermellinata di Rovigo	PER	23	X		X	
Livorno Bianca	PLB	24				
Livorno Nera	PLN	24				
Mericanel della Brianza	MER	24		X	X	
Millefiori di Lonigo	PML	23	X		X	
Modenese	MOD	24	X			
Mugellese	MUG	24		X		
Padovana Argenta	PPA	24		X	X	
Padovana Camosciata	PPC	24		X	X	
Padovana Dorata	PPD	24		X	X	
Pepoi	PPP	24		X	X	
Polverara Bianca	PPB	24			X	
Polverara Nera	PPN	24			X	
Robusta Lionata	PRL	23	X		X	
Robusta Maculata	PRM	24	X		X	
Romagnola	ROM	24		X		
Siciliana	SIC	24		X		X
Valdarnese	VLD	24	X			
Valplatani	VLP	20				X
Total		541	141	192	307	66

### Contrasting groups for comparative analyses

To identify genomic regions under selection, the breeds were categorized into contrasting groups for comparative analysis. The groups were formed according to the information available for the populations included in the dataset, including differences in live weight and geographical area of origin (Table 1). Based on live weight, populations with an average live weight more than 3.5 kg were classified as heavy and the populations with an average live weight less than 1.5 kg were classified as light. For the classification based on geography, with Italy extending from the 47th parallel in the North to the 37th parallel in the South, the populations reared in regions above the 45th parallel were classified as the Northern group and those below the 40th parallel as the Southern group. In addition, considering that all the populations included in this study are raised as backyard chickens, the 23 local Italian chicken populations were categorized as a meta-population within a single fifth group defined as “local”.

### Population structure

To investigate relationships within and between the four groups (heavy vs light and Northern vs Southern comparisons), multi-dimensional scaling (MDS) of the distance matrix was inferred using the adegenet R package [21]. Unsupervised hierarchical clustering was also carried out using the ADMIXTURE 1.23 software [22], for values of K from 3 to 5. The DISTRUCT program [23] was used to graphically display ancestry within each population.

### Identification of signatures of selection

We performed pairwise comparisons of populations for (i) heavy vs light and (ii) Northern vs Southern Italy (Table 1) Based on using the *rehh* package [24] of the R software putative signatures of selection were investigated using the *Rsb* and *XP-EHH* tests. A within-population test *(iHS*) was also computed for each of the four groups, and for the local group. For the *iHS* test, information on the ancestral and derived allele state is needed for each SNP because it is based on the ratio of the EHH associated with each allele. In our analysis, the ancestral allele was inferred as the most common allele within the 23 chicken populations. The *iHS* score for each SNP was transformed into two-sided p-value as: piHS =  − log_10_[1–2|Φ(*iHS*)− 0.5|], where Φ(*iHS*) is the cumulative Gaussian distribution function of *iHS* [24]. For the *Rsb* and *XP-EHH* tests, haplotypes were reconstructed from the genotyped SNPs using the fastPHASE 1.4 software [25]. We used the toolkit implemented in the *imputeqc* R package [26] to estimate the optimal number of haplotype clusters (K) needed for haplotype phasing. The *Imputeqc* package was designed to assess the imputation quality and/or to choose the model parameters for imputation. In our data, K = 30 provided the best imputation quality (for 5% of masked data) and was used in fastPHASE. Under the assumption that *Rsb* and *XP-EHH* values were normally distributed, a Z-test was applied to identify significant SNPs under selection. Two-sided *p*-values were derived as p*Rsb* =  − log_10_[1–2|Φ(*Rsb*)− 0.5|] and p*XP-EHH* =  − log_10_[1–2|Φ(*XP-EHH*)-0.5|], where Φ (x) is the Gaussian cumulative distribution function.

To detect signatures of selection, the 250-kb sliding windows were used with 10-kb overlaps between consecutive adjacent window. For each of the three tests, a window was classified as putatively under selection when it contained at least three SNPs that exceeded the significance threshold of − log_10_(p-value) = 4.

### Runs of homozygosity

Runs of homozygosity (ROH) were identified using the PLINK v1.9 software [20], applying a sliding-window approach to scan individual SNP genotypes and detect homozygous segments. The parameters applied to define a ROH were: (i) a sliding window of 50 SNPs across the genome; (ii) the proportion of homozygous overlapping windows was set to 0.05; (iii) the minimum number of consecutive SNPs included in a ROH was set to 100; (iv) the minimum length of an ROH was set to 1 Mb; (v) the maximum gap between consecutive homozygous SNPs was set to 1000 kb; (vi) a density of one SNP per 100 kb was set; and (vii) a maximum of two SNPs with missing genotype and up to one heterozygous genotype were allowed in a ROH. Common ROH among populations within each group were identified by counting the number of times the SNP was detected in those ROH, and dividing this value by the number of animals in each group, obtaining a locus homozygosity range. To identify ROH islands, the top 0.999 SNPs of the percentile distribution of the locus homozygosity range were selected and adjacent SNPs that met this threshold were merged into ROH islands.

### Distance-based permutational multivariate analysis of molecular variance

An analysis of molecular variance (AMOVA) was conducted to partition the between-sample genetic-distance matrix into variation due to live weight category (heavy/light) and due to geographical area (Northern/Southern Italy). Specifically, a permutational approach based on a distance matrix was followed to perform the AMOVA and test for statistical significance [27, 28], using the R implementation in the *vegan* package [29]. Based on the SNP genotypes, Hamming distances [30] between samples were calculated. The resulting D distance matrix was then partitioned as in the following models: (i) D_(n,n)_ ~ BW_(n)_, (ii) D_(n,n)_ ~ GEO_(n)_, and (iii) D_(n,n)_ ~ BW_(n)_ + GEO_(n)_, where BW and GEO are the chickens’ live body weight and geographical location, respectively, and n is the sample size. The significance of between-group differences (heavy vs light chickens, and Northern vs Southern Italy chickens) was determined based on 1000 permutations of the data by shuffling each population’s labels of heavy/light and Northern/Southern Italy.

### Gene identification and functional enrichment analysis

Genomic coordinates for all identified signatures of selection were interrogated for genes that are annotated in the *Gallus_gallus*-5.0 genome assembly. Separate lists were created for (i) genes that overlapped with the signatures of selection identified by both the *Rsb* and *XP-EHH* tests in both comparisons (i.e., heavy vs light, and Northern vs Southern Italy); and (ii) genes that overlapped with the signatures of selection identified by *iHS* for all five groups (heavy, light, local, Northern and Southern Italy). Using the online Database for Annotation, Visualization and Integrated Discovery (DAVID) software version 6.8 (https://david.ncifcrf.gov/), the gene lists were examined for significant over-representation of genes with particular functional categories. The DAVID software uses thousands of annotation terms in several annotation categories, such as Gene Ontology (GO), Biological Process, GO Molecular Function, and InterPro Domains to examine gene lists for enriched processes and functions. An adjusted Benjamini-corrected *p*-value of 0.05 was used as the criterion for statistical significance of enrichment. In addition, to investigate the biological function of each annotated gene and the phenotypes that they are known to affect, a comprehensive literature search was conducted, including information from other species.

## Results

### Population structure

Multi-dimensional scaling indicated close relationships between the populations that originated from the same geographical area (see Additional file 1: Fig. S1a and Additional file 2: Fig. S2a). In both these figures, the genetic diversity can be described as a triangle with apexes corresponding to: (1) the Siciliana (SIC) breed, (2) populations belonging to the Padovana breeds (PPA, PPC and PPD) and (3) the Robusta breeds (PRM and PRL), as reported in Cendron et al. [19]. The results also supported the separation into the two geographic groups, i.e. Northern vs Southern Italy (see Additional file 2: Fig. S2a). Moreover, no single isolated population was identified and none of the populations in each of the four groups showed marked genetic variation. There are some overlaps between a few heavy and light populations (see Additional file 1: Fig. S1a) and among the Northern populations (see Additional file 2: Fig. S2a), which indicate a close relationship and admixture for these breeds (see Additional file 1: Fig. S1b and Additional file 2: Fig. S2b). In fact, the first few ancestral components (K = 3–5) were related to geographic origin and highlight low admixture among the populations originating from the different regions. Moreover, shared ancestral components were identified between populations belonging to the same group.

### Partitioning of the genetic variance among groups

Techniques such as multivariate AMOVA can be used to determine the partitioning of the genetic diversity across different hierarchical levels such as breeds, groups of breeds, or geographical regions [31]. First, we partitioned the matrix of genetic distances by weight category (heavy/light), which explained 15.6% of the genetic variance and was significant (*p*-value < 0.001). Similar results were obtained when partitioning by geographical area (Northern/Southern Italy), which explained 14.4% of the genetic variance and was also significant (*p*-value < 0.001). The combined effect of weight and geographical area from the model D_(n,n)_ ~ BW_(n)_ + GEO_(n)_ resulted in similar variance components as obtained from the unifactorial models (weight: 24%; geographical area: 16.5%), and both were still significant (*p*-value < 0.001 in both cases). These results suggest that the weight and geographical area groupings were largely orthogonal, i.e., independent from each other. The experimental design did not allow testing of the interaction between weight and geographic area, which could have provided further insights into the relationship between them.

### Identification of signatures of selection using *iHS*

**Fig. 1:**
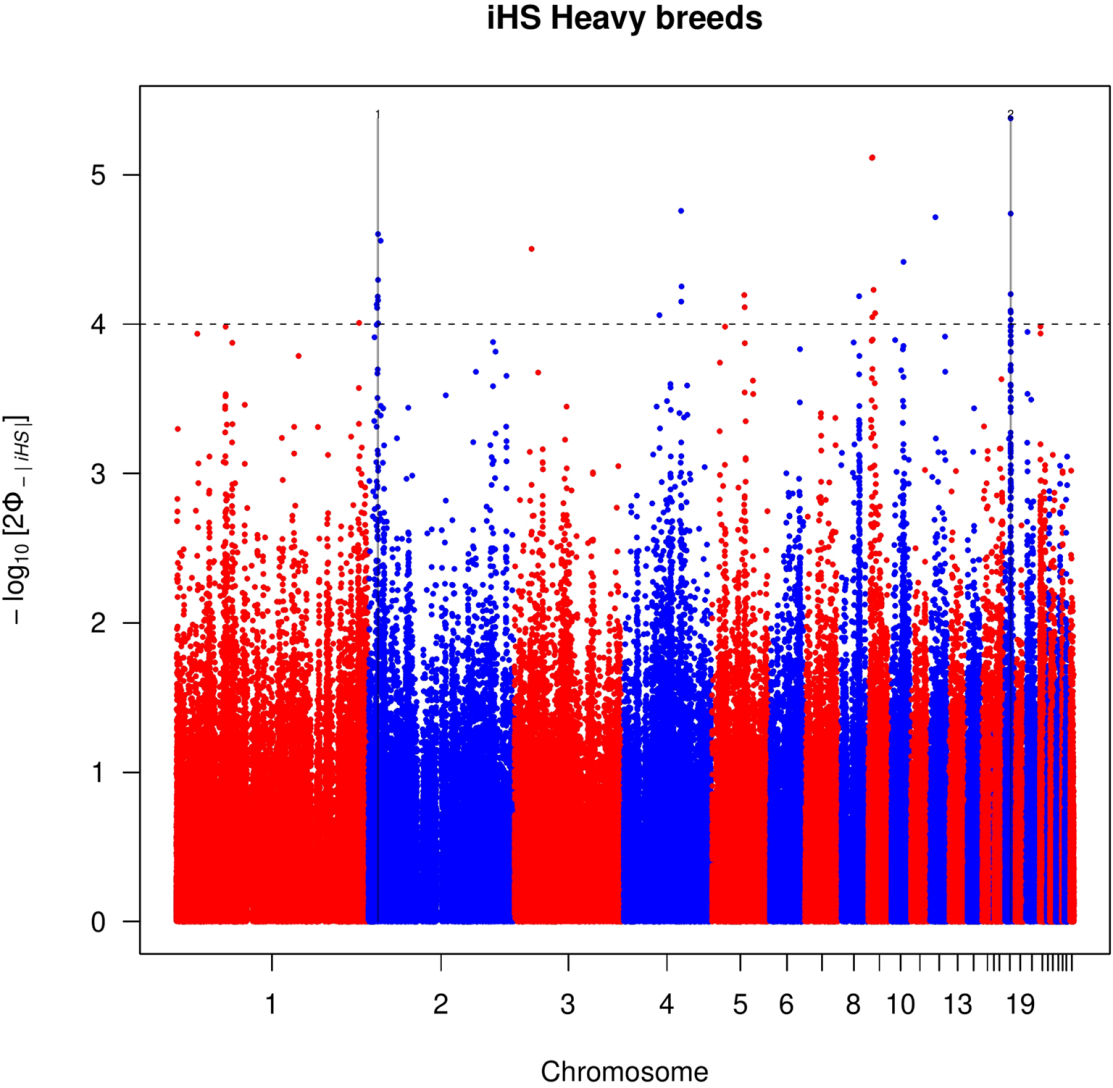
Manhattan plot of the genome-wide *iHS* analysis for the heavy chicken populations. Horizontal dashed line marks the significance threshold applied to detect the outlier SNPs [–log_10_(*p-*value) = 4]

**Fig. 2:**
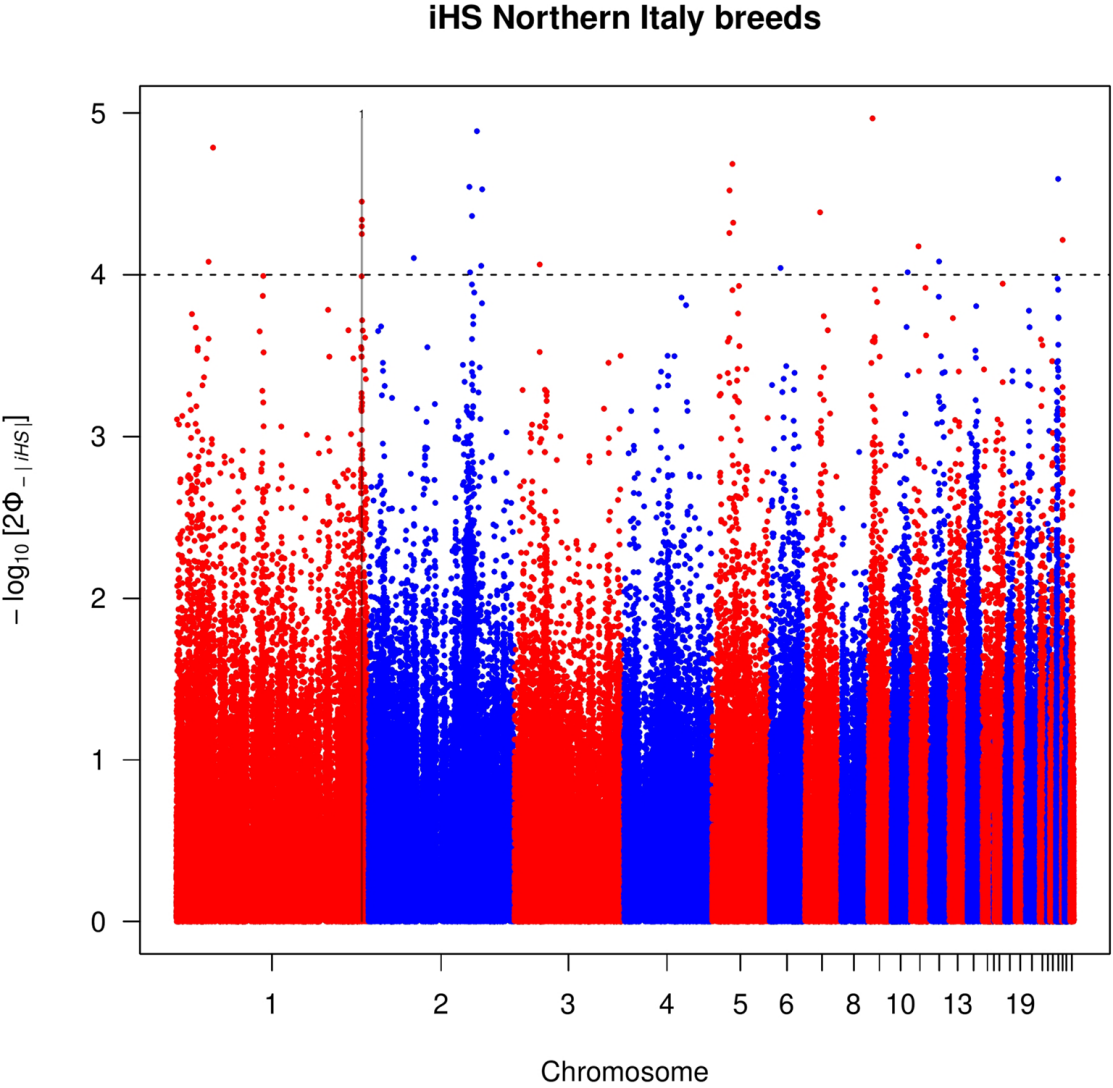
Manhattan plot of the genome-wide *iHS* analysis for the Northern Italian chicken populations. Horizontal dashed line marks the significance threshold applied to detect the outlier SNPs [–log_10_(*p-*value) = 4]

**Fig. 3:**
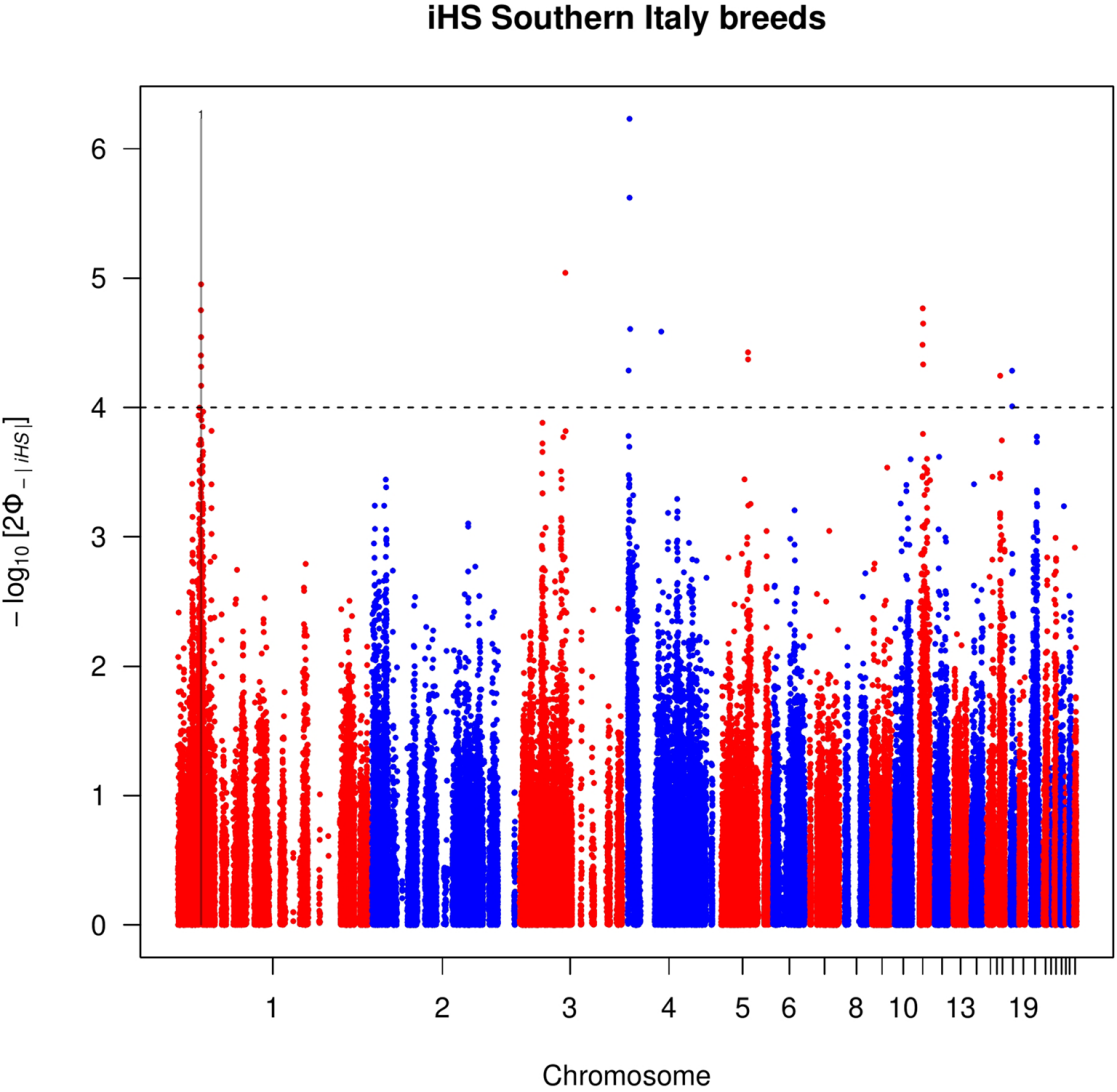
Manhattan plot of the genome-wide *iHS* analysis for the Southern Italian chicken populations. Horizontal dashed line marks the significance threshold applied to detect the outlier SNPs [–log_10_(*p-*value) = 4]

Forty-two autosomal outlier SNPs showed strong evidence of selection [− log_10_(*p*-value) > 4] in all the groups except for the populations classified as light (see Manhattan plots for each group in Figs. 1, 2, 3, and Additional file 3: Fig. S3). These outliers defined eight candidate genomic regions across seven chromosomes (GGA, *Gallus gallus* chromosome) that were putatively under positive selection and distributed: four for the local group, two for the heavy group, and one for the Northern and one for the Southern Italy groups (Table 2). These genomic regions ranged from 460 kb (on GGA8 for the local group) to 550 kb (on GGA25 for the local group). In total, 65 genes and uncharacterized genes (LOC) were located in these eight regions (Table 2).

**Table 2:** Putative signatures of selection identified in the *iHS* analysis within each of the Northern, Southern, heavy, and local population groups

Group	GGA	Start (bp)	End (bp)	Length (kb)	Genes
Northern	1	188,750,000	189,220,000	470	*FZD4, PRSS23*, *ME, FAM181B*, *PRCP*, *RAB30*, *CCDC90B*
Southern	1	24,540,000	25,050,000	510	*CTTNBP2*, *CFTR*, *GASZ*, *WNT2*, *ST7*, *ST7-OT3_2*, *CAPZA2*, *MET*
Heavy	2	10,000,000	10,490,000	490	*DIP2C*, *LARP4B*, *GTPBP4*, *WDR37*
	18^a^	6,200,000	6,710,000	510	*ANKFN1*, *NOG*, *C17orf67*, *DGKE*, *TRIM25*, *COIL*, *SCPEP1*, *RAB11FIP4*, *gga-mir-1561*, *gga-mir-193a, gga-mir-365-2*, *UTP6*, *SUZ12*, *CRLF3*, *ATAD5*
Local	4	4,870,000	5,360,000	490	*FGF13*, *F9*, *MCF2*, *ATP11C*, *ARL13A*, *XKRX*, *NOX1*, *CSTF2*, *TRMT12*, *SYTL4*, *SRPX2*, *TSPAN6*, *TNMD*, *PCDH19*
	7	16,830,000	17,310,000	480	*CHN1*, *gga-mir-1570*, *CHRNA1*, *WIPF1*, *GPR155*, *SCRN3*, *CIR1*, *SP9*, *SP3*
	8	15,270,000	15,730,000	460	-
	25	660,000	1,210,000	550	*UBQLN4*, *LAMTOR2*, *OTUD7B*, *MTMR11*, *SF3B*, *COPA*, *EDPE*, *S100A11*

*Gallus gallus* chromosome number, GGA

^a^Genomic region that overlapped with the between population tests (*Rsb* and *XP-EHH*)

### Identification of signatures of selection using *Rsb* and *XP-EHH*

**Fig. 4:**
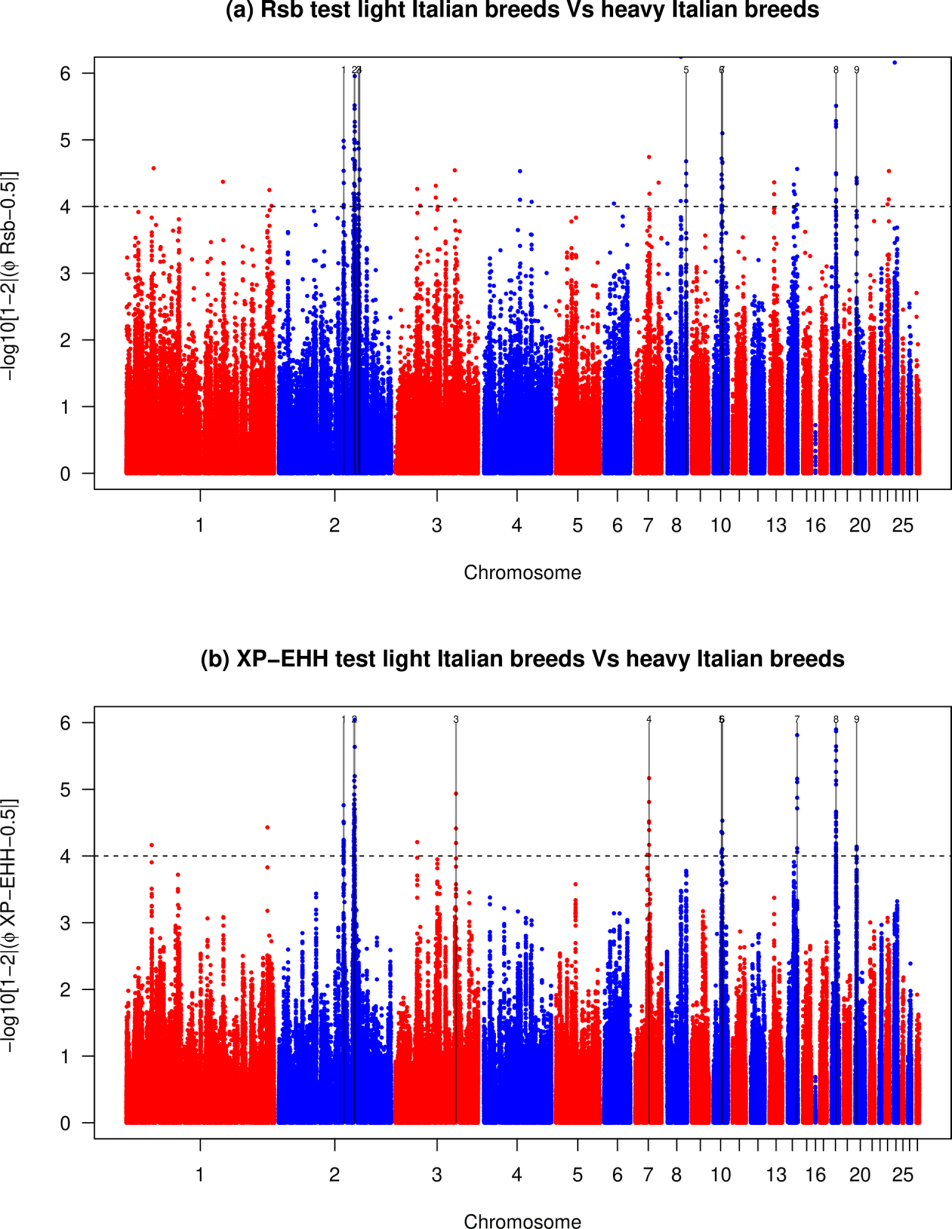
Manhattan plots of the **a** *Rsb* and **b** *XP-EHH* tests in the comparison between heavy vs light chicken populations. Horizontal dashed lines mark the significance threshold applied to detect the outlier SNPs [–log_10_(*p-*value) = 4]

**Fig. 5:**
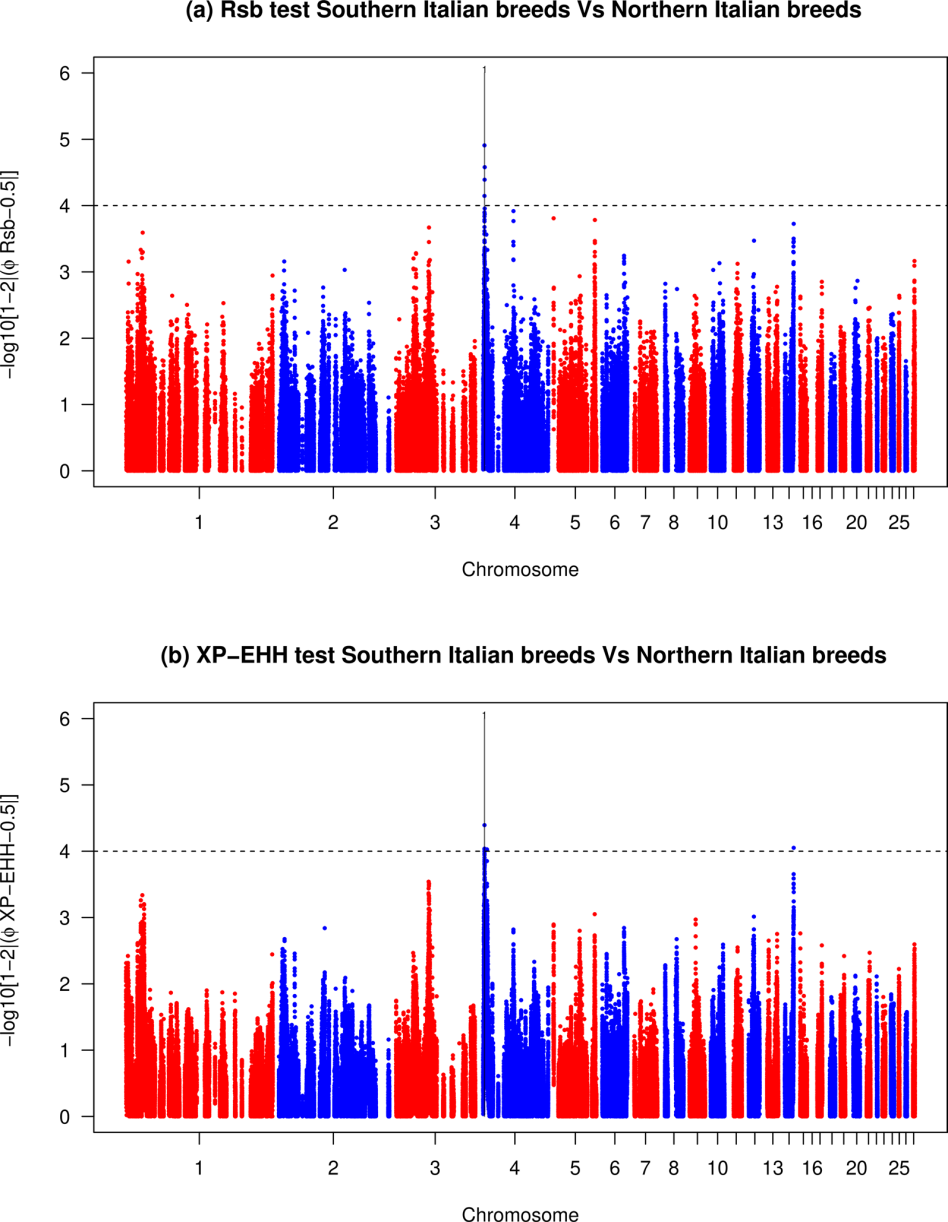
Manhattan plots of the **a** *Rsb* and **b** *XP-EHH* tests in the comparison between Northern vs Southern Italian chicken populations. Horizontal dashed lines mark the significance threshold applied to detect the outlier SNPs [–log_10_(*p-*value) = 4]

The *Rsb* test detected 73 and three SNPs that were putatively under selection for the heavy vs light (Fig. 4a) and Northern vs Southern Italy comparisons (Fig. 5a), respectively. These markers defined nine and one candidate regions for the comparisons between the heavy vs light and Northern vs Southern Italy groups, respectively (see Additional file 4: Table S1).The *XP-EHH* test identified 139 and four SNPs that were putatively under selection for the heavy vs light (Fig. 4b) and Northern vs Southern Italy comparisons (Fig. 5b), respectively, that defined nine and one candidate regions for these respective comparisons (see Additional file 5: Table S2).

### Overlapping regions identified by the EHH-derived statistics

Combining alternative approaches to detect signatures of selection has been suggested as a strategy to increase the reliability of studies on signatures of selection. Seven genomic regions, ranging from 320 kb (on GGA10 in the comparison heavy *vs* light breeds) to 1180 kb (on GGA2 in the comparison heavy *vs* light breeds), were identified by the between-population approaches (*Rsb* and *XP-EHH*; Table 3): six in the comparison heavy *vs* light and one in the comparison Northern *vs* Southern Italy. Two of these seven regions contained at least 30 SNPs above the significance threshold, providing potentially decisive evidence of selection, i.e. one on GGA2 (at position 99,700,000–100,880,000 bp, 62 SNPs with − log_10_ ≥ 4 in the *XP-EHH* test) and one on GGA18 (at position 6,130,000–6,670,000 bp, 30 SNPs with − log_10_ ≥ 4 in the *XP-EHH* test). Both these regions were identified in the comparison heavy *vs* light breeds. Importantly, the strong candidate region on GGA18 overlapped with a significant window identified in the *iHS* within-population test for the heavy group (Table 2). The seven candidate regions that were identified by at least two tests harboured 71 known genes (Table 3).

**Table 3:** Overlapping genomic regions identified by the two between-populations tests (*Rsb* and *XP-EHH*) between the Northern/Southern and between the heavy/light population groups

Contrasting groups	GGA	Start (bp)	End (bp)	Length (kb)	Genes
Northern *vs* Southern	4	4,870,000	5,200,000	330	*FGF13*, *F9, MCF2*, *ATP11C*, *ARL13A*, *XKRX*, *NOX1*, *CSTF2*, *TRMT12*
Heavy *vs* Light	2	85,920,000	86,410,000	490	*CHMP5*, *FH*, *SDHA*, *CCDC127*, *SLC6A19*, *SLC6A18*, *TERT*, *CLPTM1L*, *LPCAT1*, *NDUFS6*, *IRX4*
	2	99,700,000	100,880,000	1180	*LRRC30*, *LAMA1*, *ARHGAP28*, *AKAP7L*, *EPB41L3*, *ZBTB14*, *C18ORF42*, *DLGAP1L*
	10	11,050,000	11,370,000	320	*PDE8A*, *SCARNA15*, *FSD2*, *WHDC1*, *HOMER2, FAM103A1, C10H15ORF40, BTBD1*, *TM6SF1*, *HDGFRP3*, *BNC1*
	10	12,070,000	12,550,000	480	*TMC3*, *STARD5*, *IL16*, *C10H15ORF26*, *MESDC1*, *MESDC2*, *CEMIP*, *ABHD17C*, *ARNT2*
	18^a^	6,130,000	6,670,000	540	*ANKFN1*, *NOG*, *C18H17ORF67*, *DGKE*, *TRIM25*, *COIL*, *SCPEP1*, *RAB11FIP4*, *MIR1561*, *MIR193A*, *MIR365-2*, *COPRS*, *UTP6*, *SUZ12*
	20	2,160,000	2,510,000	350	*CHMP48*, *ZNF341*, *MIR6674*, *PXMP4*, *E2F1*, *NECAB3*, *CBFA2T2*, *SNTA1*, *TOX2*

*Gallus gallus* chromosome number, GGA

^a^Genomic region that overlapped with the within group test (*iHS*)

### Identification of signatures of selection based on regions of homozygosity

Twenty-one genomic regions that frequently appeared in a ROH were identified among all groups and are listed in Table 4. These regions ranged from 8.63 kb (on GGA4 for the light group) to 2853.42 kb (on GGA1 for the Southern Italy group). There were no overlaps between the selected regions identified with the ROH approach and those detected with the three EHH-derived statistics. Within the ROH islands, we identified several known genes and some uncharacterized genes (LOC; Table 4).

**Table 4:** Runs of homozygosity islands identified within the heavy, light, Northern, and Southern population groups

Group	GGA	Start (bp)	End (bp)	Length (kb)	Genes
Heavy	3	86,818,664	86,828,229	9.56	–
	3	86,861,928	87,641,651	779.72	*LOC107053130*, *LOC107053149*, *PRIM2*, *RAB23*, *BAG2*, *ZNF451*, *BEND6*, *DST*, *LOC107053131*, *COL21A1*
	3	87,692,868	87,767,683	74.81	*BMP5*
	4	39,754,881	39,768,758	13.87	*CENPU*, *PRIMPOL*
Light	1	16,630,326	16,645,553	15.23	*No genes*
	1	16,848,684	17,866,916	1018.32	*FAM19A5*, *LOC107051638*, *LOC107051639*
	2	97,941,997	98,028,867	86.87	*LOC107052691*
	2	98,444,389	98,584,269	139.88	*LOC107052693*, *APCDD1*, *VAPA*
	4	46,119,218	46,127,846	8.63	*AFF1*, *PTPN13*
	4	46,404,020	46,659,733	255.71	*ARHGAP24*, *COPS4*, *LIN54*, *LOC107051735*, *THAP9*, *SEC31A*
	4	46,964,555	47,158,488	193.93	*NKX6-1*, *CDS1*
Northern	3	98,743,815	99,045,750	301.93	*FAM84A*
	4	39,572,187	40,471,398	899.21	*SNX25*, *KIAA1430*, *SLC25A4*, *LOC107051755*, *LOC769128*, *HELT*, *ACSL1*, *CENPU, PRIMPOL*, *CASP3*, *LOC107051754*, *IRF2*, *ENPP6*, *STOX2*, *TRAPPC11*, *RWDD4*, *LOC107051753*, *ING2*, *CDKN2AIP*, *LOC100858888*, *LOC100858925*, *WWC2*, *DCTD*, *LOC107051751*, *TENM3*, *LOC107051752*
	5	39,968,737	40,139,698	170.96	*NRXN3*, *MIR1799*, *LOC107053526*, *LOC107053524*
	5	41,082,716	41,433,436	350.72	*-*
	11	4,048,187	4,250,262	202.08	*-*
Southern	1	2,977,765	3,214,900	237.13	*PODXL*, *MKLN1*
	1	3,258,785	6,094,202	2835.42	*LOC107054126*, *LOC418249*, *LOC107054102*, *MIR29B1*, *MIR29A*, *K123*, *IL2RA*, *RBM17*, *PFKFB3*, *LOC107053972*, *PRKCQ*, *SFMBT2*, *LOC101752189*, *LOC101751191*, *LOC107054603*, *LOC419112*, *ITIH5*, *ITIH2*, *KIN, ATP5C1*, *TAF3*, *LOC107054553*, *GATA3*, *LOC107054641*, *LOC107054627*, *LOC107054620*, *LOC107054917*, *LOC100859811*, *MIR1626*, *LOC101747941*, *CELF2, MIR1596*, *USP6NL*
	1	148,678,584	149,784,495	1105.91	*LOC107051465*, *SLITRK5*, *LOC101748664*
	3	45,812,852	47,048,955	1236.10	*LOC107053025*, *FNDC1*, *SF3B5*, *STX11*, *TRNAL-UAA*, *LOC107053022*, *LOC107053024*, *LOC107053023*, *UTRN*, *MIR1734*, *LOC107053061*, *EPM2A*, *FBXO30*, *LOC101748225*, *SHPRH*, *GRM1*, *RAB32*, *ADGB*
	11	17,818,723	18,355,368	536.64	*IRF8*, *LOC768665*, *COX4I1*, *EMC8*, *GINS2*, *GSE1*, *LOC107054326*, *LOC107054321*, *KIAA0513*, *LOC100857445*, *TRNAM-CAU*, *MAP1LC3B*, *ZCCHC14*, *JPH3*, *KLHDC4*, *SLC7A5*, *LOC107054327*, *CA5B*, *LOC107054328*, *BANP*

Chromosome number *Gallus gallus* (GGA), positions of the genomic regions (in base pairs, bp) and length are reported

### Functional annotation enrichment analysis

To better understand the biological implications, enrichment analyses were performed for the set of genes that overlapped with the signatures of selection identified by both the *Rsb* and *XP-EHH* tests in each of the two comparisons and, for the set of genes that overlapped with the candidate regions identified by the *iHS* test. The only significantly enriched annotation cluster (Benjamini-corrected *p*-value < 0.05) was observed with the *iHS* test for the local populations and included processes and pathways related to intermediate filament (GO:0005882), structural constituent of cytoskeleton (GO:0005200), and keratin (IPR003461) (see Additional file 6: Table S3).

## Discussion

To the best of our knowledge, this is the first genome-wide scan study of putative signatures of selection in local Italian chicken populations. Several factors could have led to the identification of signatures of selection in these populations, such as body weight (heavy vs light) and the geographical area of origin (Northern vs Southern Italy) (Table 1). Most local Italian chickens are Mediterranean-type breeds or populations that are known to produce eggs and meat for the rural family and/or niche products [32]. Some can be regarded as meat-type chicken breeds, including Valdarnese, Robusta Lionata, Robusta Maculata, Millefiori di Lonigo, and Ermellinata di Rovigo [33], although their productive performance is lower than that of commercial broiler lines. These local breeds have been mainly raised as backyard chickens and are, thus, more resistant to diseases and viruses than commercial chickens, for which selective breeding has reduced resistance to infectious diseases [34]. In fact, to adapt to these backyard conditions, selective sweeps might have occurred in genomic regions that are related to immune responses and disease sensitivity [35]. Effects of the geographical area of origin on genomic regions that have been subjected to selection have also been reported in local Italian goats [36] and sheep [37]. Geographical location, coupled with smallholder farm practices, likely imposed multiple environmental stressors on the studied chicken populations that may have affected their fitness and led to their adaptation to these environments over time through changes in allele frequency of beneficial or detrimental alleles.

A number of factors can affect the identification of signatures of selection, including genetic structure, population size, bottlenecks, and migration [38]. Assuming that populations with a similar structure have undergone similar evolutionary processes [39], we used populations with a high degree of within-population genetic homogeneity and shared ancestry components to detect signatures of selection [19], as was also confirmed by the MDS and Admixture results (see Additional file 1: Fig. S1 and Additional file 2: Fig. S2). We also categorized the populations into four groups for comparative analysis. In fact, including more populations in a group may identify a specific history of selection for each production purpose, instead of population-specific selection histories, which can facilitate the interpretation of the identified signatures of selection [39, 40].

To identify signatures of selection, we used different statistical methods based on the decay of haplotype homozygosity (*iHS*, *Rsb*, and *XP-EHH*) and based on regions of homozygosity (ROH). The combination of different approaches is an effective way to identify signatures of selection [38] and, together with the use of high-density SNP panels, can boost the detection accuracy and avoid unknown biases [8, 41, 42]. Moreover, we used LD-based pruning because it can account for the effects of ascertainment bias on the identification of signatures of selection, producing results that are most comparable to those obtained from whole-genome sequence data and therefore it is recommended for practical use [43, 44].

This study detected 15 genomic regions that were potentially under selection using the extended haplotype homozygosity (EHH)-derived statistics. Eight of these regions were detected within a single group (*iHS*) and seven were identified by combining the results of *Rsb* and *XP-EHH*, which revealed divergent selection between groups, thus providing good evidence that these signals are not artifacts. Twenty-one additional genomic regions were identified with the ROH approach.

There were no overlaps between the regions under selection that were identified with ROH and those detected with the three extended haplotype homozygosity (EHH)-derived statistics. This may be because ROH can detect signatures of selection related to any trait, while the heavy vs light or Northern vs Southern Italy comparisons are more likely to detect signals related to the investigated trait. Each of these statistics has its advantages and disadvantages and can capture a specific genomic region under selection [13–15]. This is not surprising as there are differences in the statistics underlying each approach for detecting the signatures of different types of selection across different timescales [14]. Moreover, the genomic regions detected by ROH can also result from other evolutionary processes, such as inbreeding, bottlenecks, and genetic drift e.g., [16, 18, 45, 46]. Therefore, considering ROH regions as signatures of selection should be viewed with caution.

Numerous genomic studies of local chicken populations worldwide have allowed the identification of signatures of selection in local breeds, using methods based either on an excess of haplotype homozygosity or deformation of the allele frequency spectrum e.g., [8, 9, 11, 35, 41]. One observation that has emerged from this study is that, in most cases, the signatures of selection detected in local chicken breeds do not overlap across studies and even between lines from the same geographical location within the same study e.g., [35]. This is mainly explained by the fact that, following their expansion through human migrations, founder populations of present-day local chicken breeds have experienced drastic bottlenecks [47]. In addition, being genetically isolated, these populations have independently evolved to adapt to diverse environmental conditions. Given that standing genetic variation is the major contributor to adaptation in chicken [48], it is not surprising that most of the signatures of selection are breed-specific because of differences in genetic background between chicken breeds.

The putative genomic regions under selection identified in our study (Tables 2, 3 and 4) spanned many candidate genes with diverse molecular and cellular functions. Therefore, in our comparison with the literature, we considered mainly the genes in the identified regions that are related to traits involved in livestock breeding. Moreover, the number of identified regions potentially under selection was larger for regions related to differences in live weight than for those related to differences in geographical area of origin (Table 3).

### Identification of signatures of selection using *iHs*

The *iHS* analysis was performed to detect recent and incomplete selective sweeps [13] within the five groups. This approach exploits information on allele frequencies of both selected and neighboring SNPs, which increases its power to detect signatures of selection [15]. This analysis is also more suited to genotyping data that are generated from SNP chips than to whole-genome sequence data, thus reducing the problems of ascertainment bias [49].

In the Northern breeds, the genes within the signature of selection on GGA1 were recently reported as putative positively selected genes related to cold adaptability in chickens [50]. In particular, the *PRCP* and *FAM181B* genes may participate in the adaptation to cold conditions by regulating angiogenesis and nervous system development [51, 52]. These genes could have a role in the adaptation of the Northern breeds to the cold conditions of their habitat region. Also, we identified the *FZD4* as a candidate gene, which is associated with the pattern of phenotypic variation of plumage color (white, mixed and brown) in chicken. Plumage color is an important qualitative trait that can serve as marker for breed identification and can be considered indirectly as an economically important trait that is under the influence of multiple genes, gene–gene interactions, and environmental factors [53]. Several local Northern breeds show a white (Bianca di Saluzzo, Polverara Bianca, Ermellinata di Rovigo) or brown plumage color (Bionda Piemontese, Robusta Lionata, Padovana Camosciata). The detected genomic regions on GGA1 for the Southern populations included candidate genes involved in thermo-tolerance and local adaptation, as for example *ST7*, which may be involved in the differences in thermo-tolerance and heat stress response mechanisms in indigenous chickens [54].

The role of the *WDR37* gene on GGA2 for the heavy group is also interesting as it encodes a member of the WD-repeat protein family that is involved in growth-related processes, including cell cycle progression and gene regulation. A previous study [55] reported that *WDR37* was differentially expressed between broilers selected for fast and slow growth. This gene has also recently been reported as a candidate for body weight in Korean native chickens [56].

Finally, in the local group that includes all populations, signatures of selection were observed in genomic regions that included genes related to meat fatty acid composition in Korean native chicken (*ATP11C*) [57], and to immune traits in chicken (*PCDH19*) [58]. Within this region, the *GPR155* gene is another candidate that is associated with high feed efficiency [59]. In a previous study [19], the identification of ROH islands within these local chickens considered as a meta-population, identified candidate genes involved in body weight and feed conversion ratio. However, there were no overlaps between the regions under selection identified here with the haplotype homozygosity approaches and those detected based on ROH analysis. The two studies agreed only on the chromosomes (GGA7 and GGA8) that hosted the selective sweeps.

### Identification of signatures of selection using *Rsb* and *XP-EHH*

The *Rsb* and *XP-EHH* tests were applied to detect potential selective sweeps that were fixed (or nearly fixed) in one group but still segregated in the other groups. Climate and farming systems vary between chicken populations from Northern and Southern Italy and between the heavy and light groups. These aspects have an impact on genome shaping in terms of regions under selection and result in differences among populations and groups [19].

The genomic region on GGA4 that was identified in the comparison between Northern and Southern Italian populations included nine candidate genes, such as *NOX1*, which plays an important role in the process of heat stress [60]. In fact, exposure of farm animals to high summer environmental temperatures, as for example in the south of Italy, negatively affects animal husbandry. Other candidate genes are involved in reproduction traits in livestock species, such as *FGF13* in chicken [61] and *MCF2* in cattle [62].

Among the candidate genes in the comparison between the heavy vs light breeds, several genes were identified on GGA2: *SLC6A19*, which is related to growth and metabolism in the Muscovy Duck [63]; *EPB41L3*, which has been reported as a promising gene for growth and meat production traits in sheep [64]; and *ZBTB14*, which is listed as a candidate gene for carcass and growth traits in chicken based on haplotype-based genome-wide association studies [10].

The largest overlap between genomic regions showing evidence of signatures of selection that was identified by the three approaches was located on GGA18 (*iHS* of heavy breeds, Table 2; *Rsb* and *XP-EHH* between heavy vs light breeds). Several genes belonging to the Noggin family were detected in this genomic region, such as the *NOG* gene, which has been suggested to be critical for normal bone and joint development [65]. Other interesting genes were also mapped to this region, such as *DGKE*, a candidate gene involved in abdominal fat deposition in chickens [66], *SCPEP1*, which has an important role in the regulation of the body and intramuscular fat content in pig [67], and *RAB11FIP4*, which is a candidate gene for body weight in American mink [68].

### Identification of signatures of selection based on regions of homozygosity

In chicken, several studies have reported that ROH regions can harbour candidate genes associated with production traits, immune response, and environmental adaptation [41, 46, 69, 70]. For the group of heavy chickens, several genes in three regions of GGA3 have been reported as candidates related to muscle growth and overlap with ROH islands detected in Italian autochthonous turkey breeds [71]: *BEND6*, which was identified as a candidate gene for intramuscular fat content in chicken [72]; *COL21A1*, which is regulated by growth factors and is involved in muscle growth [73]; and *BMP5*, which is a strong candidate gene for body size in livestock [74]. In the group of light chickens, we identified the *AFF1* gene within a ROH island on GGA4, which is known to have a lower expression level in mallards (wild ancestors with a low weight) than in Pekin ducks (large body size), and thus is related with body weight [75]. Within the ROH islands detected for the Northern group, we identified genes that are known to influence different phenotypic traits in chicken, but that are not directly linked with local adaptation, such as *SNX25*, a key gene in the regulation of TGF-β signaling and therefore, contributes to the immune system [76], or *ACSL1*, a candidate gene for fat deposition in chickens [77]. Finally, for the group of Southern chickens, the detected ROH islands hosted several interesting genes, such as: *PFKBB3*, which together with other genes belongs to the heat shock protein gene family, as a heat responsive gene [78]; genes related with pigmentation, a complex trait that is influenced by the genetic background and other factors, including the environment and endocrine factors, e.g. the *RAB32* gene, which has a crucial role in the pigmentation process, i.e. in the melanosome biogenesis, degradation, and transport, and that acts in a functionally redundant way by regulating skin melanocyte pigmentation and controlling the post-Golgi trafficking of tyrosinase (TYR) and tyrosinase-related protein 1 (TYRP1) [79]; and the *IRF8* gene, which is a critical transcriptional regulator of the innate and adaptive immune system and has been shown to have a role in the hyperpigmentation and immune development in chicken [80].

## Conclusions

In this study, we detected several putative regions containing signatures of selection and genes that differ between groups of chicken populations. These results are in line with the breeding histories of the different populations. Identification of shared signals by different methods can provide persuasive evidence about the effect of selection on these specific regions. Since the genes that exhibit signatures of selection are related to local adaptation, we can hypothesize that positive selection in Italian chicken populations may have been driven by the need to survive in a backyard environment. Although the candidate genes were identified using different statistical methods, they may include some false positives. With the development of additional genomic approaches and experimental technologies, additional genes are likely to be found.

## Supplementary Information

**Additional file 1: Figure S1.** Results on the population structure between heavy vs light chicken populations. (A) multidimensional scaling; (B) unsupervised hierarchical clustering. For a full definition of populations, see Table 1.

**Additional file 2: Figure S2.** Results on the population structure between Northern vs Southern Italian chicken populations. (A) multidimensional scaling; (B) unsupervised hierarchical clustering. For a full definition of populations, see Table 1.

**Additional file 3: Figure S3.** Manhattan plot of the genome-wide *iHS *analysis for all the local chicken populations. Horizontal dashed line marks the significance threshold applied to detect the outlier SNPs [–log10(*p-*value) = 4].

**Additional file 4: Table S1.** Genomic regions identified using *Rsb* statistic in the comparisons between the heavy vs light and Northern vs Southern Italy chicken populations.

**Additional file 5: Table S2.** Genomic regions identified using the *XP-EHH* statistic in the comparisons between the heavy vs light and Northern vs Southern Italy chicken populations.

**Additional file 6: Table S3.** Functional annotation clustering results for the candidate genes observed in the *iHS* test of the local populations. Significantly enriched functional term clusters (Benjamin-corrected p-value < 0.05) are in bold.

## Data Availability

The datasets generated and/or analyzed during the current study are not publicly available but are available from the corresponding author upon reasonable request.
