# Supplementary figures and images for: Genome-wide mapping of signatures of selection using a high-density array identified candidate genes for growth traits and local adaptation in chickens
Source: Genet Sel Evol. 2023 Mar 23;55:20. doi: 10.1186/s12711-023-00790-6 (PMC10035218; doi:10.1186/s12711-023-00790-6)

a)

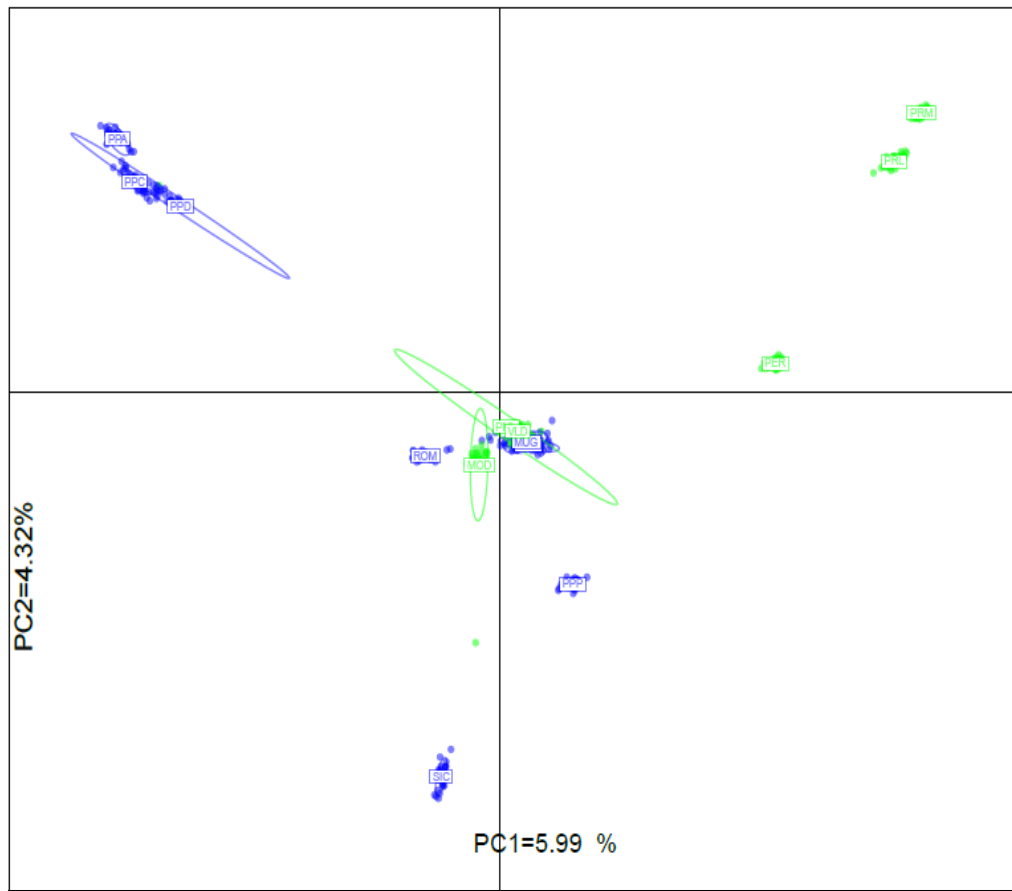

b)

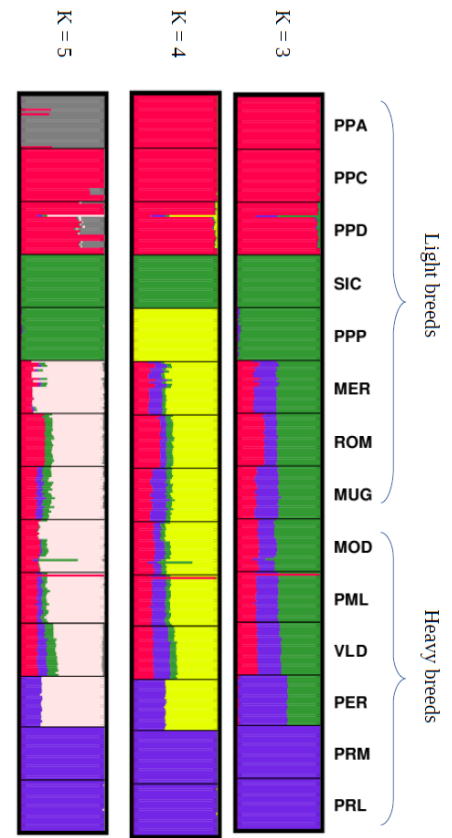

Supplement: Supplementary file 1 — Additional file 1: Figure S1. Results on the population structure between heavy vs light chicken populations. (A) multidimensional scaling; (B) unsupervised hierarchical clustering. For a full definition of populations, see Table 1. [file 12711_2023_790_MOESM1_ESM.pdf]

a)

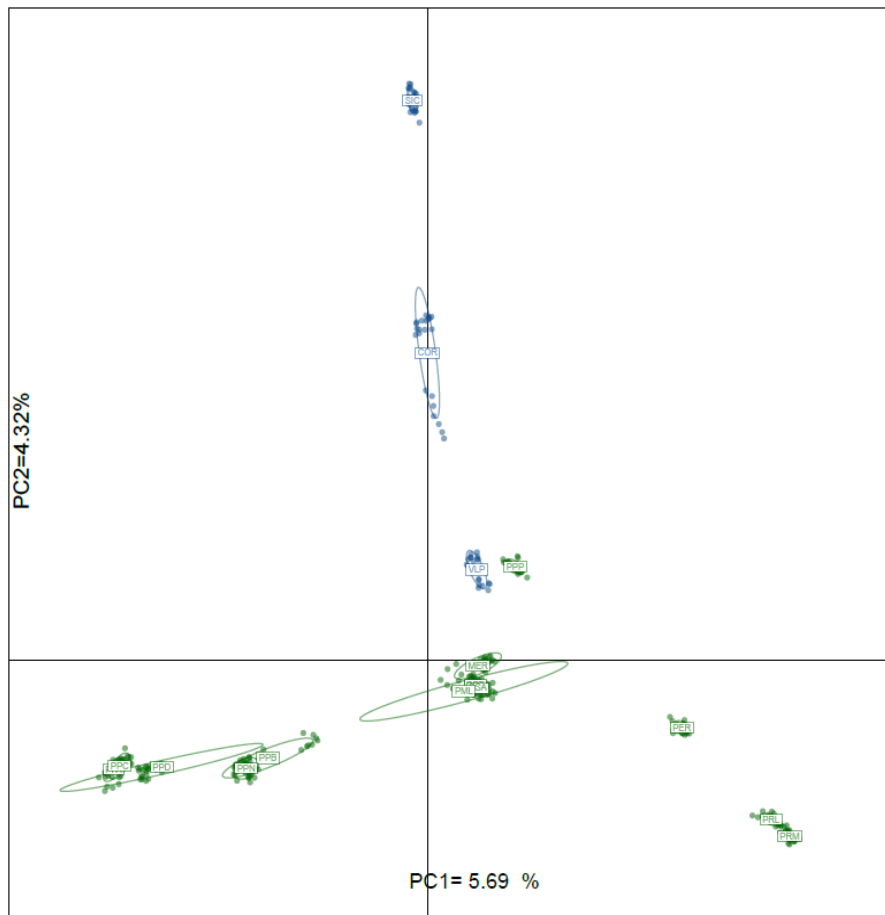

b)

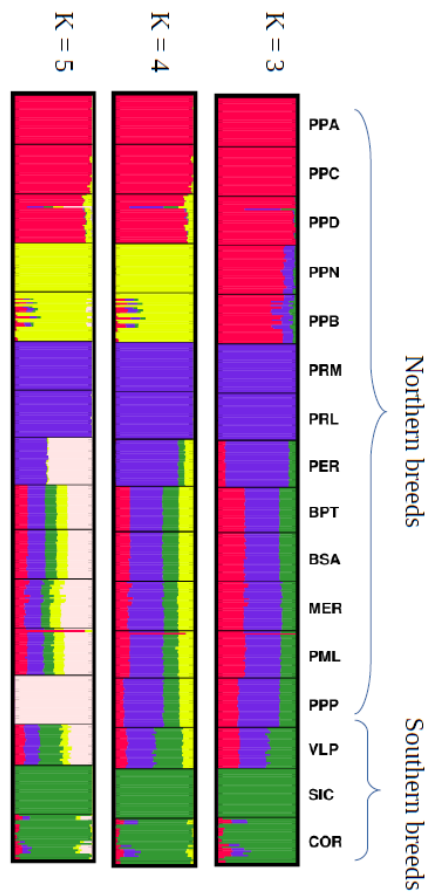

Supplement: Supplementary file 2 — Additional file 2: Figure S2. Results on the population structure between Northern vs Southern Italian chicken populations. (A) multidimensional scaling; (B) unsupervised hierarchical clustering. For a full definition of populations, see Table 1. [file 12711_2023_790_MOESM2_ESM.pdf]

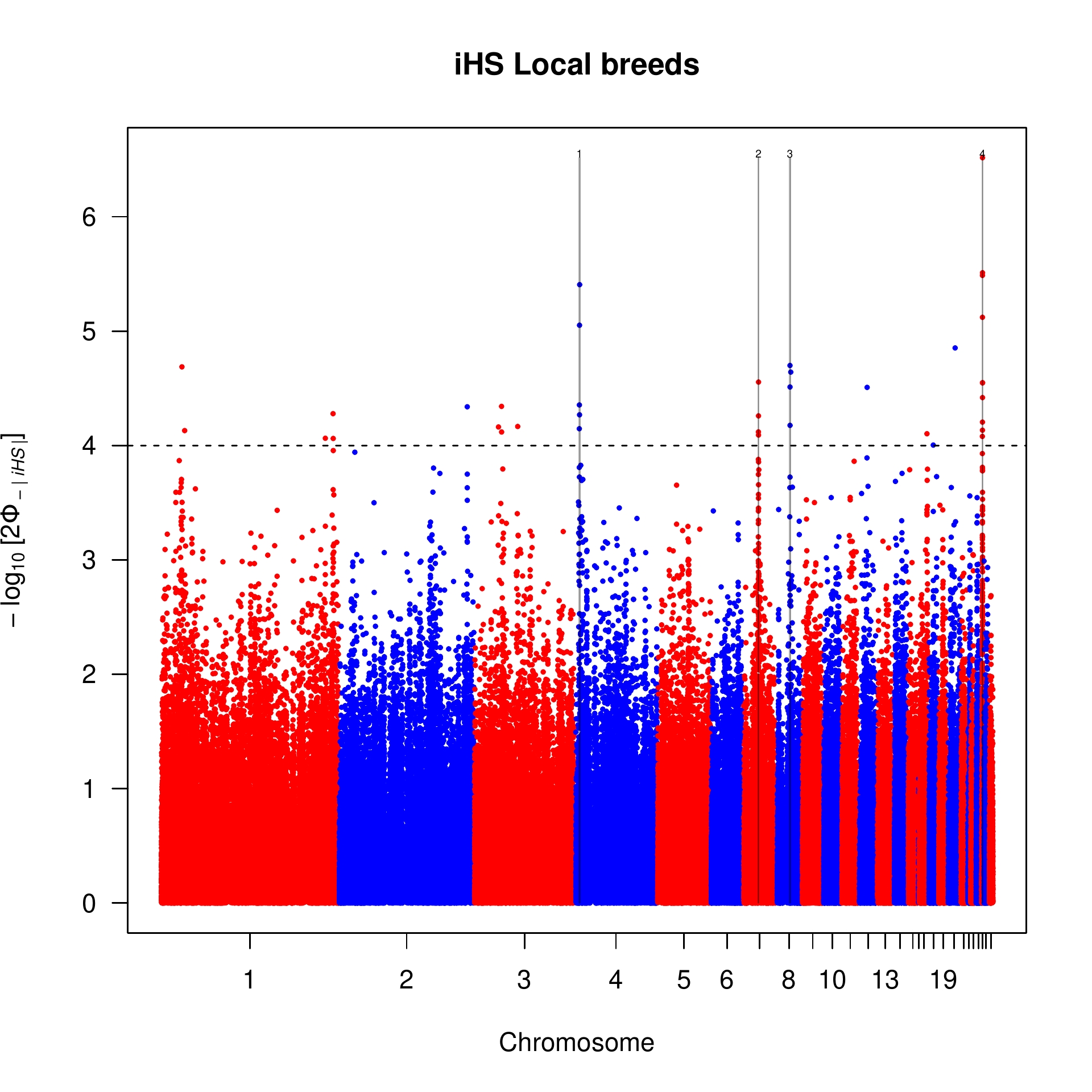

Supplement: Supplementary file 3 — Additional file 3: Figure S3. Manhattan plot of the genome-wide iHS analysis for all the local chicken populations. Horizontal dashed line marks the significance threshold applied to detect the outlier SNPs [–log10(p-value) = 4]. [file 12711_2023_790_MOESM3_ESM.jpg]
