# Supplementary material for: Genome-wide mapping of signatures of selection using a high-density array identified candidate genes for growth traits and local adaptation in chickens
Source: Genet Sel Evol. 2023 Mar 23;55:20. doi: 10.1186/s12711-023-00790-6 (PMC10035218; doi:10.1186/s12711-023-00790-6)
Supplement: Supplementary file 4 — Additional file 4: Table S1. Genomic regions identified using Rsb statistic in the comparisons between the heavy vs light and Northern vs Southern Italy chicken populations. [file 12711_2023_790_MOESM4_ESM.doc]

**Additional file 4: Table S1**. Genomic regions identified using *Rsb* statistic in the comparisons between heavy *vs* light and Northern *vs* Southern Italy chicken breeds.

| **Contrasting groups** | **Region** | **GGA** | **Start (bp)** | **End (bp)** | **N_MRK** | **N_Mrk exceeding the threshold** |
| --- | --- | --- | --- | --- | --- | --- |
| Heavy *vs* Light | 1 | 2 | 85920000 | 86410000 | 99 | 5 |
|  | 2 | 2 | 99700000 | 100940000 | 428 | 30 |
|  | 3 | 2 | 105630000 | 106120000 | 156 | 4 |
|  | 4 | 2 | 106840000 | 107260000 | 169 | 3 |
|  | 5 | 8 | 26570000 | 27060000 | 272 | 5 |
|  | 6 | 10 | 11050000 | 11560000 | 339 | 6 |
|  | 7 | 10 | 12070000 | 12570000 | 309 | 6 |
|  | 8 | 18 | 6130000 | 6690000 | 374 | 11 |
|  | 9 | 20 | 2160000 | 2510000 | 183 | 3 |
| Northern *vs* Southern | 1 | 4 | 4870000 | 5200000 | 122 | 3 |
