# Supplementary material for: Genome-wide mapping of signatures of selection using a high-density array identified candidate genes for growth traits and local adaptation in chickens
Source: Genet Sel Evol. 2023 Mar 23;55:20. doi: 10.1186/s12711-023-00790-6 (PMC10035218; doi:10.1186/s12711-023-00790-6)
Supplement: Supplementary file 5 — Additional file 5: Table S2. Genomic regions identified using the XP-EHH statistic in the comparisons between the heavy vs light and Northern vs Southern Italy chicken populations. [file 12711_2023_790_MOESM5_ESM.doc]

**Additional file 5: Table S2**. Genomic regions identified using *XP-EHH* statistic in the comparisons between heavy *vs* light and Northern *vs* Southern Italy chicken breeds.

| **Contrasting groups** | **Region** | **GGA** | **Start (bp)** | **End (bp)** | **N_MRK** | **N_Mrk exceeding the threshold** |
| --- | --- | --- | --- | --- | --- | --- |
| Heavy *vs* Light | 1 | 2 | 85690000 | 86440000 | 171 | 20 |
|  | 2 | 2 | 99310000 | 100880000 | 498 | 62 |
|  | 3 | 3 | 79900000 | 80390000 | 106 | 3 |
|  | 4 | 7 | 18920000 | 19420000 | 106 | 7 |
|  | 5 | 10 | 10880000 | 11370000 | 319 | 4 |
|  | 6 | 10 | 12060000 | 12550000 | 301 | 3 |
|  | 7 | 14 | 13670000 | 14160000 | 210 | 7 |
|  | 8 | 18 | 6020000 | 6670000 | 426 | 30 |
|  | 9 | 20 | 2120000 | 2520000 | 214 | 3 |
| Northern *vs* Southern | 1 | 4 | 4720000 | 5200000 | 178 | 4 |
