# Supplementary material for: Genome-wide mapping of signatures of selection using a high-density array identified candidate genes for growth traits and local adaptation in chickens
Source: Genet Sel Evol. 2023 Mar 23;55:20. doi: 10.1186/s12711-023-00790-6 (PMC10035218; doi:10.1186/s12711-023-00790-6)
Supplement: Supplementary file 6 — Additional file 6: Table S3. Functional annotation clustering results for the candidate genes observed in the iHS test of the local populations. Significantly enriched functional term clusters (Benjamin-corrected p-value < 0.05) are in bold. [file 12711_2023_790_MOESM6_ESM.doc]

**Additional file 6: Table S3. Functional annotation clustering results for candidate genes observed in the *iHS* test of local breeds. Significantly enriched functional term clusters (Benjamin-corrected p-value < 0.05) are in bold.**

| **Annotation Cluster 1** | **Enrichment Score: 8.28** |  |  |  |  |  |  |  |  |  |  |  |
| --- | --- | --- | --- | --- | --- | --- | --- | --- | --- | --- | --- | --- |
| *Category* | *Term* | *Count* | *%* | *p-value* | *Genes* | *List Total* | *Pop Hits* | *Pop Total* | *Fold Enrichment* | *Bonferroni* | *Benjamin* | *FDR* |
| INTERPRO | IPR003461:Keratin | 8 | 24.24 | **8.64E-10** | ENSGALG00000026987, ENSGALG00000024138, ENSGALG00000028843, ENSGALG00000024136, ENSGALG00000027640, ENSGALG00000024141, ENSGALG00000029144, ENSGALG00000028706 | 27 | 88 | 11164 | 37.58 | 7.26E-08 | **7.26E-08** | 7.26E-08 |
| GOTERM  CC DIRECT | GO:0005882  intermediate filament | 8 | 24.24 | **6.73E-09** | ENSGALG00000026987, ENSGALG00000024138, ENSGALG00000028843, ENSGALG00000024136, ENSGALG00000027640, ENSGALG00000024141, ENSGALG00000029144, ENSGALG00000028706 | 26 | 111 | 10057 | 27.87 | 2.76E-07 | **2.76E-07** | 2.76E-07 |
| GOTERM MF DIRECT | GO:0005200  structural constituent of cytoskeleton | 8 | 24.24 | **2.54E-08** | ENSGALG00000026987, ENSGALG00000024138, ENSGALG00000028843, ENSGALG00000024136, ENSGALG00000027640, ENSGALG00000024141, ENSGALG00000029144, ENSGALG00000028706 | 23 | 132 | 8532 | 22.48 | 7.12E-07 | **7.12E-07** | 7.12E-07 |
|  |  |  |  |  |  |  |  |  |  |  |  |  |
| **Annotation Cluster 2** | **Enrichment Score: 0.26** |  |  |  |  |  |  |  |  |  |  |  |
| *Category* | *Term* | *Count* | *%* | *p-value* | *Genes* | *List Total* | *Pop Hits* | *Pop Total* | *Fold Enrichment* | *Bonferroni* | *Benjamin* | *FDR* |
| UP KEYWORDS | Membrane | 9 | 27.27 | 0.46 | ENSGALG00000006707, ENSGALG00000006808, ENSGALG00000009301, ENSGALG00000006822, ENSGALG00000006821, ENSGALG00000006623, ENSGALG00000006637, ENSGALG00000009153, ENSGALG00000014209 | 28 | 3177 | 11626 | 1.17 | 1 | 1 | 1 |
| UP KEYWORDS | Transmembrane helix | 8 | 24.24 | 0.52 | ENSGALG00000006707, ENSGALG00000006808, ENSGALG00000009301, ENSGALG00000006822, ENSGALG00000006821, ENSGALG00000006623, ENSGALG00000006637, ENSGALG00000014209 | 28 | 2891 | 11626 | 1.14 | 1 | 1 | 1 |
| UP KEYWORDS | Transmembrane | 8 | 24.24 | 0.52 | ENSGALG00000006707, ENSGALG00000006808, ENSGALG00000009301, ENSGALG00000006822, ENSGALG00000006821, ENSGALG00000006623, ENSGALG00000006637, ENSGALG00000014209 | 28 | 2899 | 11626 | 1.14 | 1 | 1 | 1 |
| GOTERM CC DIRECT | GO:0016021  integral component of membrane | 6 | 18.18 | 0.73 | ENSGALG00000006707, ENSGALG00000006822, ENSGALG00000006821, ENSGALG00000006623, ENSGALG00000006637, ENSGALG00000014209 | 26 | 2357 | 10057 | 0.98 | 1 | 1 | 1 |
